# Differences in metavirome among *Aedes albopictus*, *Culex tritaeniorhynchus*, and *Anopheles sinensis* in Jiangxi Province, China

**DOI:** 10.1186/s13071-025-07195-y

**Published:** 2026-01-05

**Authors:** Xin Ran, Dajin Xiao, Yangbowen Wu, Yong Shi, Shiwen Liu, Yu Bai, Qiang Zhang, Lan Liu, Qian Liu, Jianxiong Li, Minghui Zhao

**Affiliations:** 1 Laboratory of Viral Infectious Disease, The Key Laboratory of Important and Emerging Viral Infectious Diseases of Jiangxi Health Commission, Jiangxi Provincial Center for Disease Control and Prevention, Nanchang, China; 2 Jiangxi International Travel Healthcare Center, Nanchang, China

**Keywords:** Metavirome, *Ae. albopictus*, *Cx. tritaeniorhynchus*, *An. sinensis*

## Abstract

**Background:**

Mosquito metavirome research aims to comprehensively characterize the diversity of mosquito-associated viruses, particularly focusing on insect-specific viruses (ISVs) and their potential interactions with arboviruses of public health concern. Advances in next-generation sequencing (NGS) have significantly expanded our understanding of the viromic complexity within mosquito populations, revealing numerous novel viral species and genera. These studies not only contribute to viral taxonomy and evolutionary biology but also provide critical insights into the ecological dynamics between mosquitoes and their viromes.

**Methods:**

NGS was employed to characterize the metavirome of three epidemiologically significant mosquito vectors, *Aedes albopictus*, *Culex tritaeniorhynchus*, and *Anopheles sinensis* in Jiangxi Province, China. This study integrated bioinformatic workflows to conduct comparative analyses of viral composition and biological significance.

**Results:**

An analysis of the metavirome of three mosquito species in Jiangxi Province revealed 86 viruses. Of these, 49 belonged to 19 established families, while the remaining 37 were unclassified. The unclassified viruses had the highest relative abundance. The known virus families with relatively high abundances among the three mosquito species were: *Solemoviridae*, *Xinmoviridae*, *Phasmaviridae*, *Flaviviridae*, *Rhabdoviridae*, *Peribunyaviridae* and *Orthomyxoviridae*. Although the Shannon and Simpson diversity indices showed no significant differences between the three species (*p* > 0.05), substantial compositional divergence was observed in the “top 30 viruses.” The most frequently detected viruses in the *Ae. albopictus* population include High Island virus, Usinis virus, Sichuan mosquito sobemo-like virus, Guangzhou sobemo-like virus, *Barstukas virus*, Piry virus (PIRYV), *Aedes* *flavivirus* (AEFV), and *Aedes albopictus* *anphevirus* (AealbAV). The most frequently detected viruses in the *Cx. tritaeniorhynchus* population include *Hubei mosquito virus 2*, *Quang Binh virus* (QBV), *Culex tritaeniorhynchus* *rhabdovirus* (CTRV), *Yongsan sobemo-like virus 1 *(YSLV1), *Bat sobemovirus* (BSV), *Wuhan Mosquito Virus 2* (WMV2), and *Culex pseudovishnui* *bunya-like virus* (CPBV). The most frequently detected viruses in the *An. sinensis* population include *Hubei reo-like virus 12*, *Xincheng mosquito virus* (XCV), *Wuhan mosquito virus 1 *(WMV1), and *Wuhan mosquito virus 5* (WMV5).

**Conclusions:**

The most frequently detected virus profiles of the three most important mosquito species for epidemiology in Jiangxi Province, *Ae. albopictus*, *Cx. tritaeniorhynchus*, and *An. sinensis*, exhibit evident differences. Further validation of the biological characteristics, pathogenicity, vector competence, and host relationships of the identified viruses (including ISVs) is required to gain a comprehensive understanding of their roles in host–virus interactions. This will provide theoretical support for vector control efforts in Jiangxi Province.

**Graphical Abstract:**

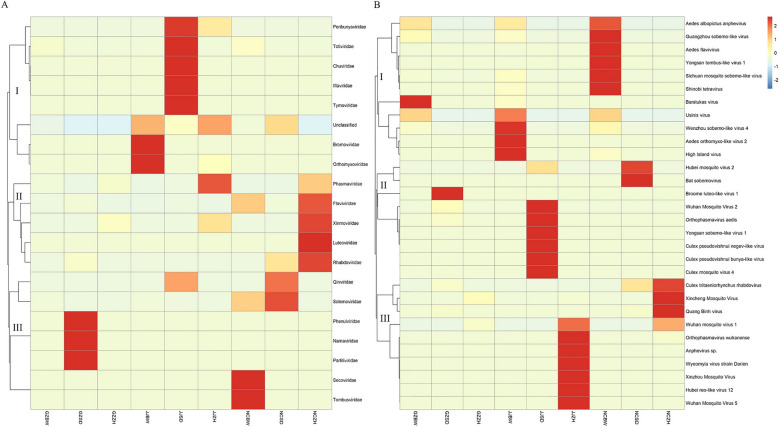

**Supplementary Information:**

The online version contains supplementary material available at 10.1186/s13071-025-07195-y.

## Background

Situated in the subtropical humid monsoon climate zone along the southern bank of the middle-lower Yangtze River, Jiangxi Province provides optimal thermohydrometric conditions for mosquito proliferation and the transmission cycles of arboviruses [1, 2]. *Aedes albopictus*, *Cx. tritaeniorhynchus*, and *An. sinensis* are common vector mosquitoes in the province. *Aedes albopictus* serves as a vector for the dengue virus (DENV), Zika virus (ZIKV), and Chikungunya virus (CHIKV), which have caused widespread infectious disease outbreaks worldwide; such outbreaks affect the health and lives of hundreds of millions of people every year [3, 4]. *Culex tritaeniorhynchus* is the primary vector for Japanese encephalitis virus (JEV), an endemic disease pathogen in Asia. This vector is prevalent in tropical and subtropical regions of East Asia and causes nearly 70,000 infections of JEV each year, which results in a mortality rate of 20–30% [5, 6]. Although *An. sinensis* is recognized as the primary vector of malaria parasites in China, other pathogens have recently been detected in it, including ZIKV, JEV, and *Getah virus* [7–10]. The first local outbreak of dengue fever occurred in Zhanggong District, Ganzhou City, Jiangxi Province, in 2017, whereas a number of municipalities and counties saw local outbreaks of the same disease in 2019 [11]. For 2 months, a total of 81 cases had been reported. Although no fatalities were recorded, the outbreak caused serious public health concern for the local people [12]. In 2021, Wang et al. reported the first detection of the ZIKV in *Cx. tritaeniorhynchus* and *An. sinensis* mosquitoes collected in Jiangxi Province [7]. Currently, *Ae. albopictus* is the only vector species that carries the DENV and ZIKV in Jiangxi Province. No study reported *Cx. tritaeniorhynchus* and *An. sinensis* transmitting these viruses [13]. While the detection of ZIKV is crucial for control measures, it can also lead to public panic and misallocation of resources if not managed effectively.

The development of next-generation sequencing (NGS) technology enabled the identification of a growing number of viruses in mosquito metagenome studies. These viruses include common pathogens, such as the yellow fever virus (YFV) [14], DENV [15], CHIKV [16], JEV [17], and ZIKV [18]. They also comprise a number of insect-specific viruses (ISVs) and unclassified viruses [19, 20]. Pathogenic insect-borne viruses pose a crucial threat to humans worldwide and place a substantial economic burden on governments. With the intensive growth of global transportation systems and the gradual urbanization of arthropods, arboviruses are emerging and spreading faster and over a wider geographical area [21]. ISVs naturally infect mosquitoes and can replicate in their cells in vitro; notably, they do not replicate in vertebrate cells nor infect humans and other vertebrates [22]. However, several ISVs also play a role in the replication and transmission of arboviruses and the development of gene expression platforms and corresponding vaccines [23, 24]. The *Culex* ISV *Bamaga virus* interfered with in vitro replication and in vivo transmission of the pathogenic flavivirus *West Nile virus* (WNV) [25, 26]. The *Nhumirim virus* (NHUV) reduced the replication of the WNV, *Saint Louis encephalitis virus*, and JEV when cocultured in the C6/36 cell line [27, 28]. NHUV also significantly inhibited the replication of the ZIKV and *dengue virus 2* in C6/36 cells and reduced the infection rate and transmissibility of ZIKV in *Ae. Aegypti* [29]. Cell-fusing agent virus and *Phasi Charoen-like virus* in *Ae. aegypti* cells affects the growth of the ZIKV, which leads to considerable changes in the cell permissivity to arbovirus infection [30]. *The Eilat virus* (EILV) can affect the replication of coinfected arboviruses, such as the CHIKV in the *Ae. albopictus* cell line and *Ae. aegypti* populations [31]. In addition, the EILV-based chimeras can be used to express arboviruses proteins, such as those from the CHIKV. These chimeric viruses can then be utilized to immunize mice against respective arboviruses [32]. EILV–CHIKV chimeric viruses offer invaluable biosafety advantages for the development of diagnostic reagents [33]. Mapping of mosquito viromes and understanding their composition and roles are crucial for gaining insights into the transmission of arboviruses and vaccine development.

A review of literature revealed the recently rapid increase in the number of published studies on the mosquito metagenome. These studies covered at least 128 mosquito species belonging to 14 genera. Their main focuses were the *Culex*, *Aedes*, and *Anopheles* genera [19]. Another study revealed *Flaviviridae*, *Rhabdoviridae*, and *Peribunyaviridae* as the three most abundant mosquito virus families, with *Flaviviridae* distributed in mosquitoes on all continents studied and with the highest detection rates in *Culex*, *Aedes*, and *Anopheles* mosquitoes [19, 34]. This work used NGS and integrated bioinformatics methods to analyze the metavirome profiles of *Ae. albopictus*, *Cx. tritaeniorhynchus*, and *An. sinensis*, which were collected in Jiangxi Province in 2023. The aim was to compare the metavirome profiles of these species and to identify potential pathogenic viruses as well as ISVs that may influence vector competence and pathogen transmission.

## Methods

### Mosquito collection

**Fig. 1:**
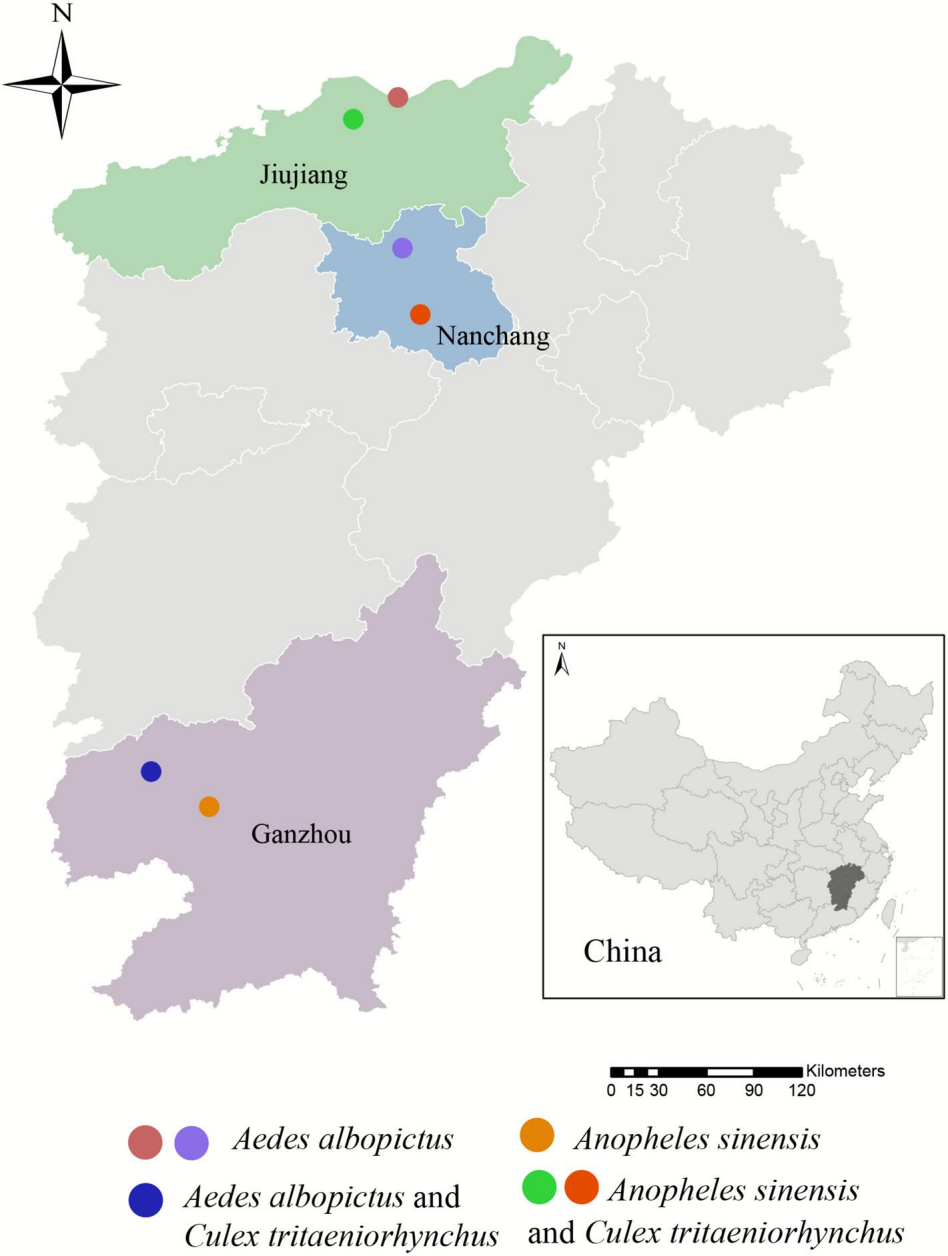
Sampling locations map of *Ae. albopictus*, *Cx. tritaeniorhynchus*, and *An. sinensis* populations

Field collections of *Ae. albopictus*, *Cx. tritaeniorhynchus*, and *An. sinensis* were conducted across three representative municipalities in Jiangxi Province (Nanchang, Ganzhou, and Jiujiang) in August 2023. The Electric Handheld Mosquito Trap (catalog no. DX3V) was used for collecting adult mosquitoes. The captured adult mosquitoes (excluded blood-fed female mosquitoes) underwent cryoanesthesia using dry ice or exposure to a laboratory freezer (−20 ℃), after which they were identified at the species level using morphological keys [35]. The species-confirmed female mosquitoes (*n* = 334) were grouped into nine taxon-specific pools (20–60 individuals per pool) on the basis of the collection site and were then stored at −80 ℃ in cryovials for subsequent analysis. Sampling locations and ecological characteristics are presented in Table 1 and shown in Fig. 1.

**Table 1:** Sampling locations and RNA quality of *Ae. albopictus*, *Cx. tritaeniorhynchus*, and *An. sinensis* populations

Cities	Code	Species	Total number	Longitude/latitude	Average temperature (2023) (℃)	Average relative humidity (2023) (%)	Average precipitation (2023) (mm)
Nanchang	NCBW	*Ae. albopictus*	33	115° 55′ 39″ E/28° 52′ 35″ N			
	NCSD	*Cx. tritaeniorhynchus*	52	116° 01′ 49″ E/28° 29′ 52″ N	19.6	72.3	1,848
	NCZH	*An. sinensis*	42	116° 01′ 49″ E/28° 29′ 52″ N			
	JJBW	*Ae. albopictus*	31	115° 54′ 08″ E/29° 43′ 41″ N			
Jiujiang	JJSD	*Cx. tritaeniorhynchus*	40	115° 39′ 04″ E/29° 36′ 20″ N	18.5	77.5	1,251
	JJZH	*An. sinensis*	25	115° 39′ 04″ E/29° 36′ 20″ N			
	GZBW	*Ae. albopictus*	26	114° 50′ 05″ E/25° 42′ 43″ N			
Ganzhou	GZSD	*Cx. tritaeniorhynchus*	40	114° 50′ 05″ E/25° 42′ 43″ N	20.2	75.0	1,542
	GZZH	*An. sinensis*	45	114° 30′ 15″ E/25° 54′ 35″ N

### Nucleic acid extraction and quality control

Frozen mosquito specimens were retrieved from −80 ℃ storage and transferred to sterile grinding tubes. Each tube received 600 µl saline solution and 6–8 zirconium-oxide grinding beads. The tubes were securely capped and homogenized at 5,000 revolutions per minute (rpm) for 45 s using a bead mill homogenizer. Following centrifugation at 12,000*g* for 10 min at 4 ℃, the supernatants were subjected to RNA extraction using a QIAamp Viral RNA Mini Kit (no. 52904) as per the manufacturer’s protocol. DNase treatment to deplete genomic DNA from the RNA extracts was performed using the Turbo DNA-free kit (Thermo Fisher Scientific) following the manufacturer’s instructions. The purified RNA was quantified using a Qubit 4 Fluorometer (Thermo Fisher Scientific) with RNA HS Assay Kit (Invitrogen, Q32855) and archived at −80 ℃ in nuclease-free microtubes.

### Library construction and sequencing

First, viral RNA enrichment was performed. RNA enrichment was performed using the Macro & Micro-test pathogen RNA enrichment platform. Probes that bind to ribosomal RNA (rRNA) were used to capture host rRNA. This was then digested with RNase H, after which DNase I was used to digest the probes and DNA. The remaining RNA was then purified.

Second, fragmentation, reverse transcription, and library construction were performed. Enriched RNA samples were purified and recovered using magnetic beads before fragmentation to ~300 base pairs (bp). Reverse transcription using random primers synthesized the first strand of complementary DNA (cDNA). The complementary strand was synthesized concurrently with end repair and A-tailing to yield the second strand of cDNA. Sequencing adapters were then ligated to both ends of the cDNA fragments. Following adapter ligation, fragment selection was performed via magnetic bead purification (0.6–0.8× bead selection). The target fragment library obtained through magnetic bead purification and selection was then enriched and purified again using magnetic beads to yield a library ready for sequencing.

Finally, the MGISEQ-2000 was used for sequencing. After library quantification, the sequencing libraries were pooled according to the required data volume. The pooled libraries then underwent ligation, digestion, and purification to obtain ligation products. These were then processed through rolling circle amplification to generate DNA nanoballs (DNBs). The DNBs were loaded onto BGI sequencing chips using the MGIDL-200 and tested on the MGISEQ-2000 sequencer (BGI Genomics). On the MGISEQ-2000 platform, PE150 sequencing (150 bp paired-end) was performed using dual-indexing for multiplex library pooling. Each library is projected to yield approximately 10 million reads.

### Data analysis

Raw sequencing reads underwent quality control via FastQC (v0.12.1; https://www.bioinformatics.babraham.aCx.uk/projects/fastqc/), followed by adapter trimming and low-quality base removal using Trimmomatic (v0.39; http://www.usadellab.org/cms/?page=trimmomatic). Clean reads were subjected to host-genome depletion against the reference genomes of *Ae. albopictus* (GCF_035046485.1), *Cx. tritaeniorhynchus* (GCA_964187845.1), and *An. sinensis* (GCA_000441895.2) using Kneaddata (v0.12.0; https://huttenhower.sph.harvard.edu/kneaddata). Host-depleted reads were assembled de novo using MEGAHIT (v1.2.9; k-mer range 21–141; https://github.com/voutcn/megahit). The resulting contigs (> 500 bp) were compared against the National Center for Biotechnology Information (NCBI) nt database via BLASTnt (v2.13.0+), with an *e*-value cutoff of 1 × 10^−5^. Further species identification was carried out on the basis of the annotation results. Viral abundance was calculated on the basis of the number of reads obtained through sequencing. The relative abundance (%) in our study was calculated using the formula: (number of annotated reads for a specific contig/total number of annotated reads) × 100. The α-diversity (Shannon and Simpson) indices were calculated using the “vegan” package in R, which has been widely used for data analysis [36].

The assembled short sequences were further assembled using MEGAHIT (v1.2.9). Near-full-length viral genomes (> 90% coverage) were identified through BLASTnt alignment against the NCBI database. Maximum-likelihood phylogenetic trees were reconstructed in MEGA (v11.0.13; https://www.megasoftware.net/) using 1000 bootstrap replicates under the best-fit substitution model.

## Results

### Sampling and sequencing results

Field collections across three municipalities in Jiangxi Province (Nanchang, Ganzhou, and Jiujiang) yielded 334 adult female mosquitoes of *Ae. albopictus*, *Cx. tritaeniorhynchus*, and *An. sinensis* (Table 1). These nine pools were then subjected to metagenomic sequencing and generated high-quality data. Each sample pool generated a data volume exceeding 20 Gb, with an average Q30 score of over 97% (Additional file 1: Supplementary Table S1). The detection and quantification of viruses could be influenced by RNA extraction, stability, integrity, and reverse transcription. Therefore, future studies should control for these variables to reach definitive conclusions.

### Virus diversity and host analysis

**Fig. 2:**
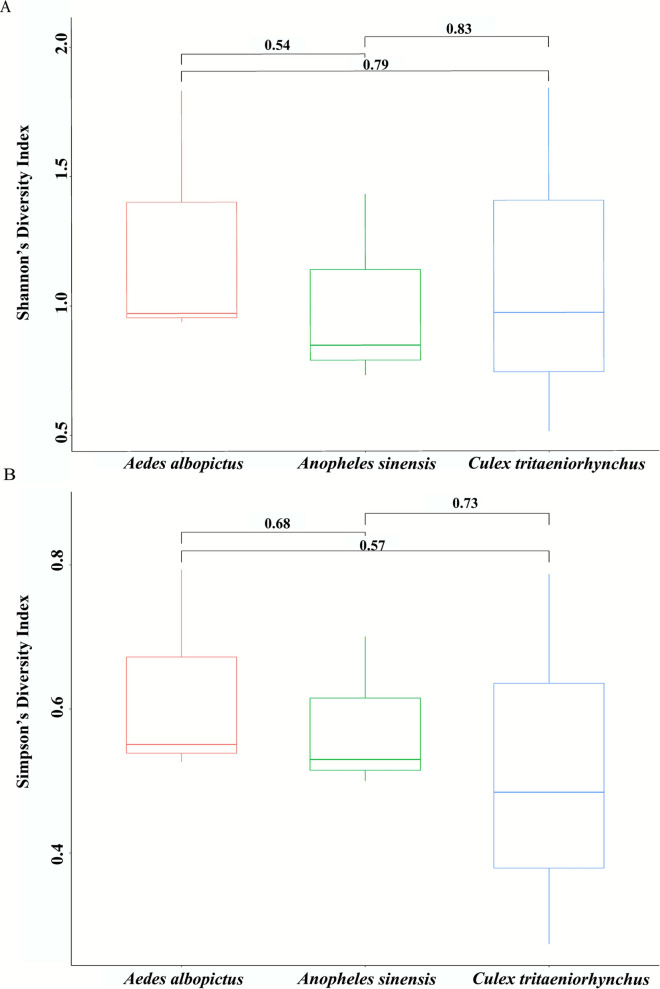
Shannon and Simpson diversity of 86 viruses in *Ae. albopictus*, *Cx. tritaeniorhynchus*, and *An. sinensis* pools

Metavirome assembly of nine mosquito pools identified 86 viruses, which comprised 49 viruses classified within 19 established families and 37 unclassified viruses (Additional file 2: Supplementary Table S2). The *Ae. albopictus* pools were assembled and annotated to 10 families (*n* = 37 total viruses), the *Cx. tritaeniorhynchus* pools to 14 families (*n* = 37 total viruses), and the *An. sinensis* pools to 7 families (*n* = 20 total viruses). Analysis of the α-diversity of these 86 viruses among the three mosquito species revealed no significant differences (Shannon and Simpson diversity *P* > 0.05; Fig. 2). Of these viruses, 43.02% (37/86) were unclassified viruses and 37.21% (32/86) were ISVs. Notably, the annotated viruses included two human–invertebrate dual-host viruses, namely, *Guapiacu virus* (GUAPV) [37] and *Wyeomyia virus strain Darien *(WVD) [38].

### Relative abundance and cluster analysis of viruses

**Fig. 3:**
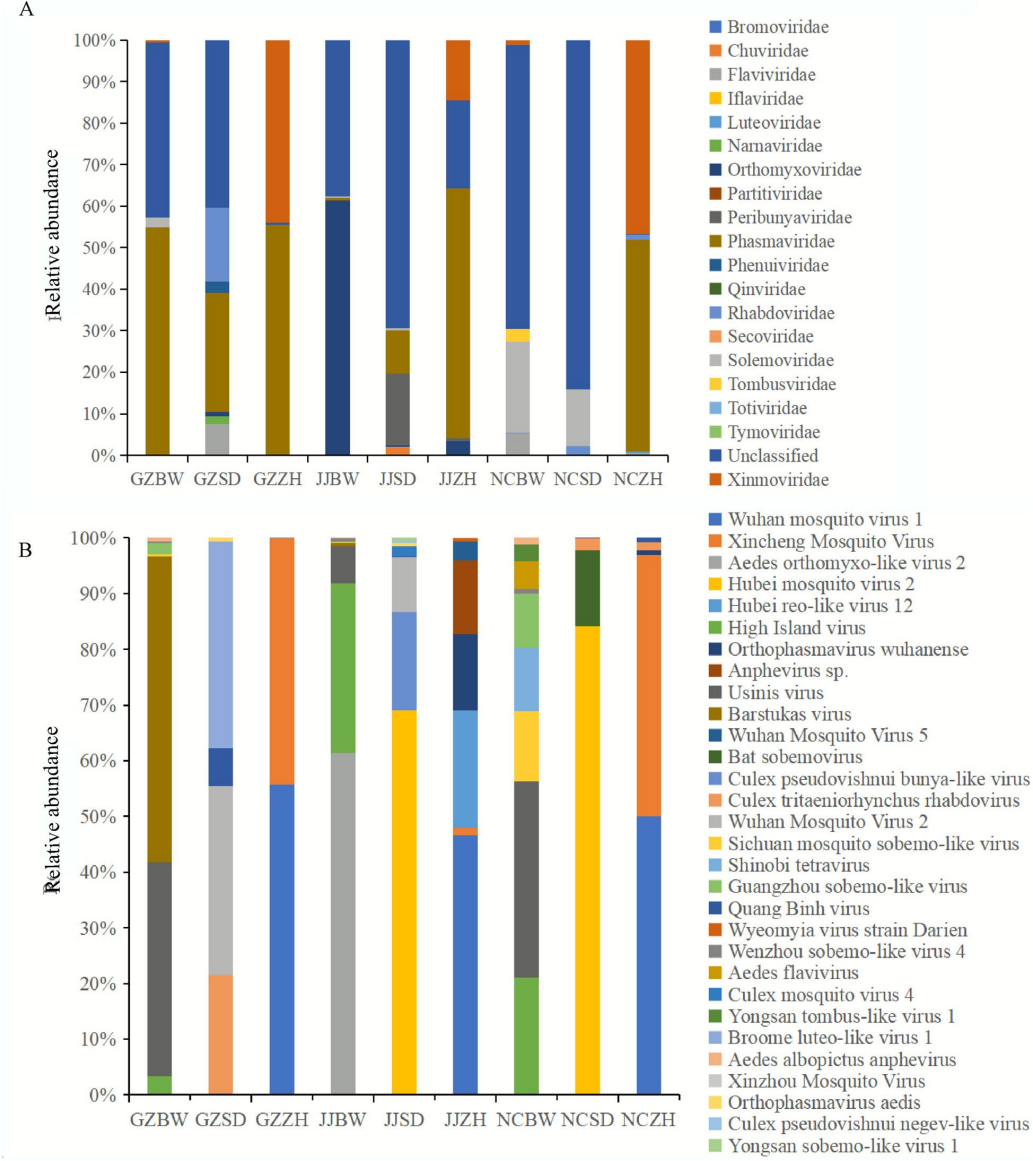
The relative abundance of viral families in *Ae. albopictus*, *Cx. tritaeniorhynchus*, and *An. sinensis* pools (**A** family level; **B** species levels top 30)

Hierarchical clustering of viral abundance profiles revealed significant compositional divergence of viruses among the nine mosquito pools at the family and species levels (top 30). At the family level (Fig. 3A), unclassified viruses were present in all nine mosquito pools, with the highest relative abundance observed in the NCSD (84.17%), JJSD (70.72%), NCBW (68.38%), and GZSD (40.38%) pools. *Phasmaviridae* constituted the predominant viral family in the NCZH (50.95%), JJZH (60.25%), GZZH (55.42%), and GZBW (54.82%) pools, whereas *Orthomyxoviridae* dominated the JJBW pool (61.64%). Notably, *Xinmoviridae* exhibited an elevated abundance in the NCZH (46.78%) and GZZH (43.92%) pools compared with the other pools. At the species level (top 30) (Fig. 3B), *Wuhan mosquito virus 1 *(WMV1) dominated all *An. sinensis* pools but was undetectable in the *Ae. albopictus* and *Cx. tritaeniorhynchus* pools. Furthermore, the *Xincheng mosquito virus* (XCV) and *Hubei reo-like virus 12* were only detected in *An. sinensis*. XCV was most abundant in the GZZH (43.92%) and NCZH (46.78%) pools and WMV1 in the JJZH (60.25%) pool. In the three *Cx. tritaeniorhynchus* pools, *Hubei mosquito virus 2* had the highest relative abundance in the NCSD (84.10%) and JJSD (70.25%) pools but was also detected in one *An. sinensis* pool (NCZH). *Broome luteo-like virus 1* was the most abundant in the GZSD (30.66%) pool. *Wuhan mosquito virus 2* (WMV2) was only detected in the three *Cx. tritaeniorhynchus* pools, and it was least abundant in the NCSD (14 reads) pool. Of the three *Ae. albopictus* pools, *Aedes orthomyxo-like virus 2* (AOMV-2) was only detected in the JJBW (61.41%) pool. Usinis virus was the most abundant in the NCBW (34.89%) pool and present at relatively high levels in the GZBW (38.39%) pool. *Barstukas virus* was the most abundant in the GZBW (54.82%) pool and was also detected in the JJBW pool. Eight viruses were detected only in the *Ae. albopictus* pools (High Island virus, Usinis virus, Sichuan mosquito sobemo-like virus, Guangzhou sobemo-like virus, *Wenzhou sobemo-like virus 4* [WSLV4], *Aedes albopictus* anphevirus (AealbAV), *Longgang virus*, and *Guato virus*).

**Fig. 4:**
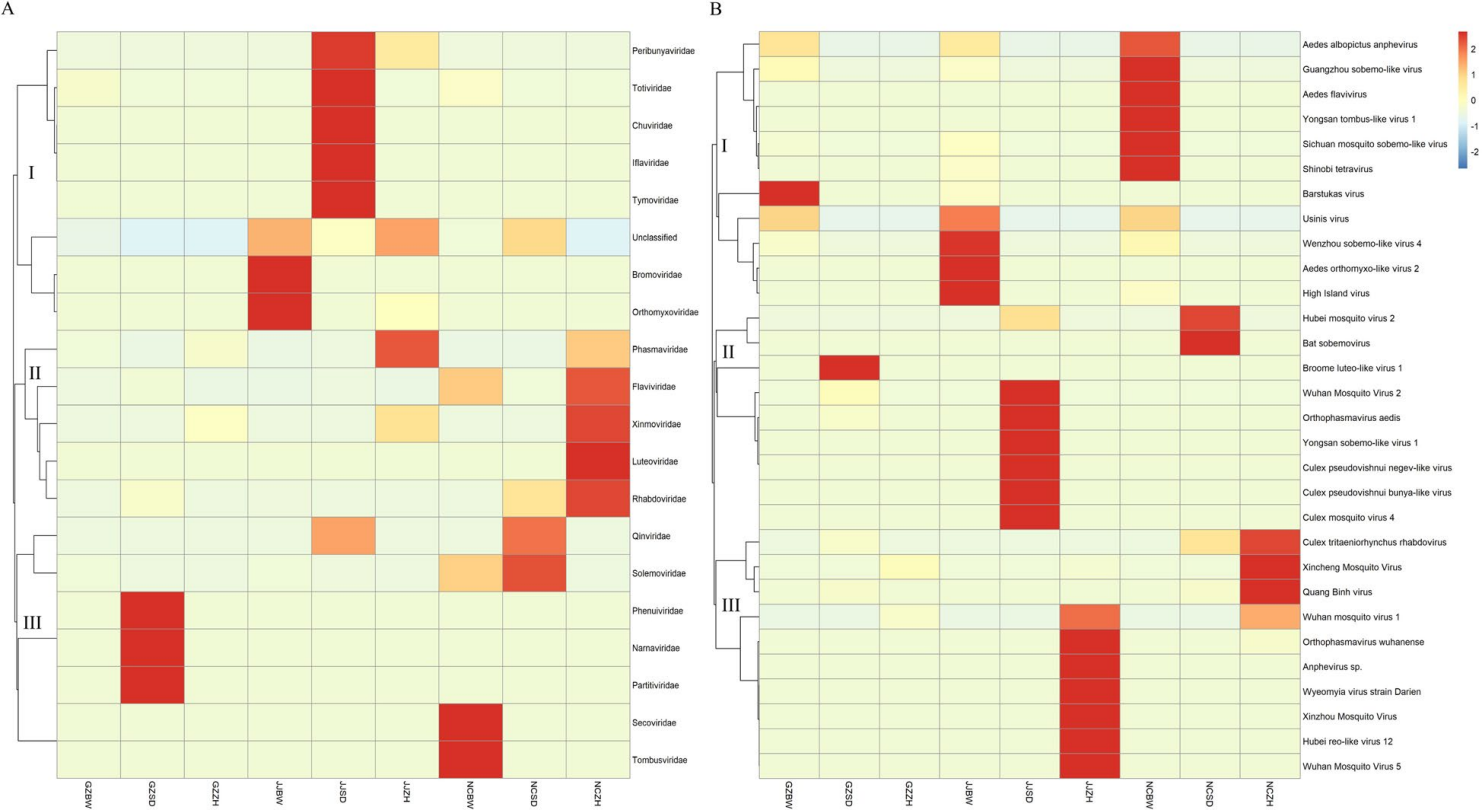
The heat map of viral families in *Ae. albopictus*, *Cx. tritaeniorhynchus*, and *An. sinensis* pools (**A** family level; **B** species levels top 30)

At the family level in the heat map (Fig. 4A), the 20 viral families (including unclassified viruses) can be divided into three major genetic branches. The unclassified viruses in the first genetic branch were closely related to *Bromoviridae* and *Orthomyxoviridae* families. At the species level (Fig. 4B), the top 30 viruses can also be divided into three genetic branches. Branch I contained 11 viruses that were relatively abundant in three *Ae. albopictus* pools. Branch II contained nine viruses that were relatively abundant in three *Cx. tritaeniorhynchus* pools. Branch III contained ten viruses that were relatively abundant in three *An. sinensis* pools.

### Complete viral genome assembly and analysis

Further de novo assembly and reconstruction yielded 17 complete viral genomes and nine RNA-dependent RNA polymerase (RdRp) genes (Table 2). The 17 complete viral genomes revealed four viruses from the *Ae. albopictus* pools: AealbAV (OR715784 and OR729834), *Shinobi tetravirus* (SHTV, PQ295851, and PQ295852), *San Gabriel mononegavirus* (PQ295853 and PQ295854), and *Longgang virus* (PQ305976). Three viruses were found in *Cx. tritaeniorhynchus* pools: *Quang Binh virus* (QBV, PQ376979, and PQ376980), *Culex pseudovishnui* *negev-like virus* (PQ376981), and *Culex tritaeniorhynchus* *rhabdovirus* (CTRV, PQ305975, and PQ305977). Four viruses were found in the *An. sinensis* pools: CTRV (PQ305978), XCV (PQ376982 and PQ376983), *Hubei virga-like virus 23* (PQ376984), and *Riboviria* sp. (PQ442368).

**Table 2:** Information of concatenated viral sequences from *Ae. albopictus*, *Cx. tritaeniorhynchus*, and *An. sinensis* populations in the present study

Mosquito	Code	Viruses	Gene types	GenBank numbers
*Ae. albopictus*	JJBW	*Aedes albopictus* *anphevirus*	Complete genome	OR715784
	JJBW	*Shinobi tetravirus*	Complete genome	PQ295851
	JJBW	*San Gabriel mononegavirus*	Complete genome	PQ295853
	JJBW	*Longgang virus*	Complete genome	PQ305976
	JJBW	*Barstukas virus*	RdRp, complete cds	PQ793244
	GZBW	*Aedes albopictus* *anphevirus*	Complete genome	OR729834
	GZBW	*Barstukas virus*	RdRp, complete cds	PQ793243
	NCBW	*Aedes albopictus* *anphevirus*	RdRp, complete cds	PQ480189
	NCBW	*San Gabriel mononegavirus*	Complete genome	PQ295854
	NCBW	*Shinobi tetravirus*	Complete genome	PQ295852
*Cx. tritaeniorhynchus*	GZSD	*Culex tritaeniorhynchus* *rhabdovirus*	Complete genome	PQ305975
	GZSD	*Quang Binh virus*	Complete genome	PQ376979
	GZSD	*Orthophasmavirus aedis*	RdRp, complete cds	PQ793245
	JJSD	*Isahaya Culex iflavirus*	RdRp, complete cds	PQ442367
	JJSD	*Quang Binh virus*	Complete genome	PQ376980
	JJSD	*Culex pseudovishnui* *negev-like virus*	Complete genome	PQ376981
	JJSD	*Orthophasmavirus aedis*	RdRp, complete cds	PQ793246
	NCSD	*Culex tritaeniorhynchus* *rhabdovirus*	Complete genome	PQ305977
*An. sinensis*	NCZH	*Culex tritaeniorhynchus* *rhabdovirus*	Complete genome	PQ376978
	NCZH	*Xincheng mosquito virus*	Complete genome	PQ376982
	GZZH	*Xincheng mosquito virus*	Complete genome	PQ376983
	GZZH	*Hubei virga-like virus 23*	Complete genome	PQ376984
	GZZH	*Riboviria* sp.	Complete genome	PQ442368
	GZZH	*XiangYunbunya-arena-like_virus 14*	RdRp, complete cds	PQ442370
	GZZH	*Wuhan mosquito virus 1*	RdRp, complete cds	PQ480190
	JJZH	*Xinzhou mosquito virus*	RdRp, complete cds	PQ442369

cds stands for ‘coding sequence’

**Fig. 5:**
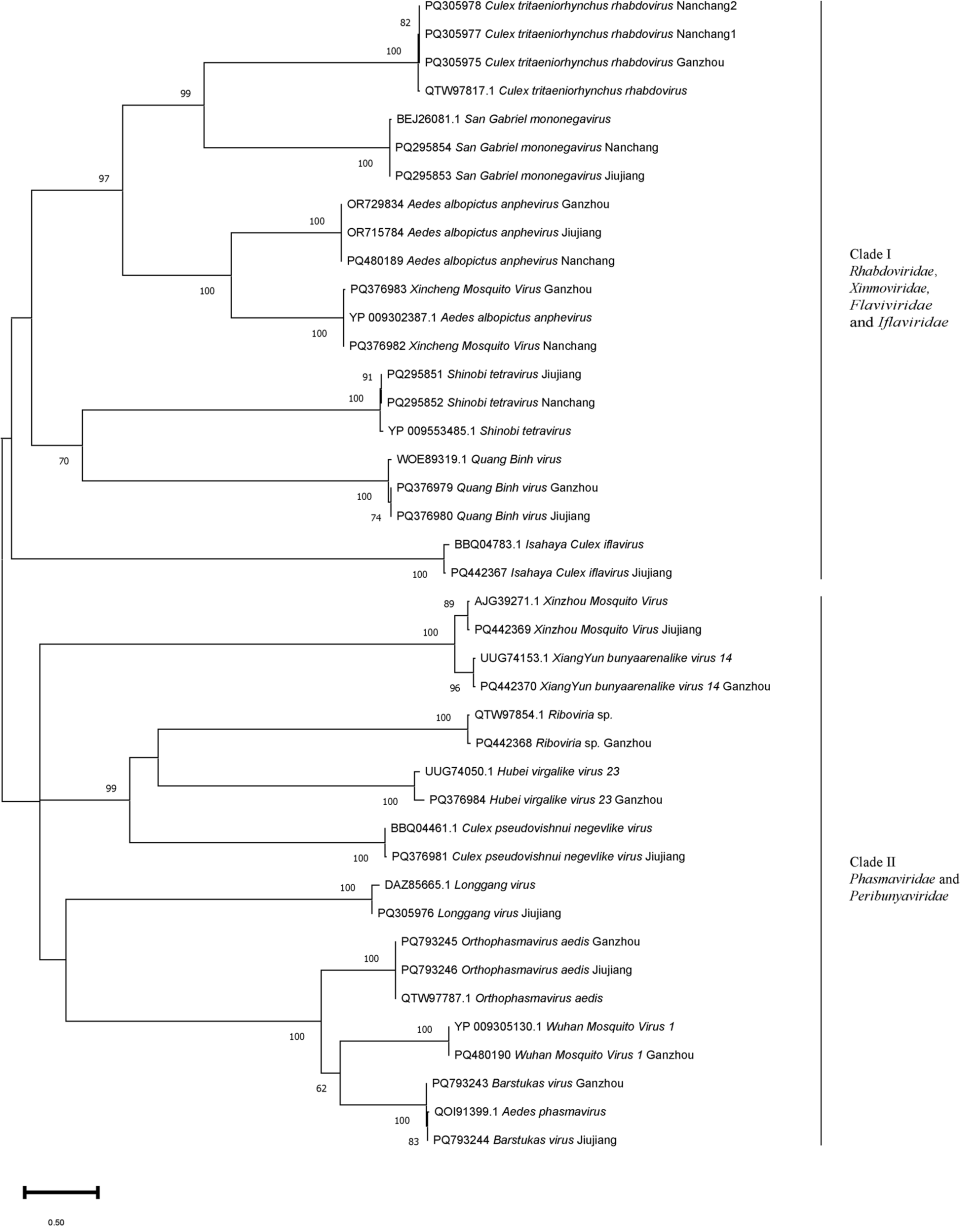
Maximum likelihood phylogeny of RdRp in 26 viruses (1,000 bootstrap replicates)

Phylogenetic reconstruction of RdRp amino acid sequences from the 26 viruses revealed two major clades (I–II; Fig. 5) under the maximum likelihood criterion (1000 bootstrap replicates). Clade I contained three CTRV (*Rhabdoviridae*; PQ305975, PQ305977, and PQ305978), three AealbAV (*Xinmoviridae*; OR715784, OR729834, and PQ480189), two XCV (*Xinmoviridae*; PQ376982 and PQ376983), two QBV (*Flaviviridae*; PQ376979 and PQ376980), *Isahaya Culex iflavirus* (*Iflaviridae*; PQ442367), and two unclassified viruses: *San Gabriel mononegavirus* (PQ295853 and PQ295854) and SHTV (PQ295851 and PQ295852). Clade II included *Xinzhou mosquito virus* (*Peribunyaviridae*; PQ442369), *Phasmaviridae* members, namely, *Orthophasmavirus aedis* (PQ793245 and PQ793246), WMV1 (PQ480190), and *Barstukas virus* (PQ793243 and PQ793244), as well as five unclassified viruses, *Culex pseudovishnui* *negev-like virus* (PQ376981), *Hubei Virga-like virus 23 *(PQ376984), *Longgang virus* (PQ305976), *Riboviria* sp. (PQ442368), and *XiangYun bunya-arena-like virus 14* (PQ442370).

## Discussion

Global surveillance of known mosquito-borne viruses has intensified following recurrent arboviral outbreaks across six continents. Yet, untargeted metaviromics has uncovered an expanding universe of mosquito-associated viruses, which is profoundly reshaping our understanding of virome diversity and evolution [34]. Here, metaviromic sequencing and computational biology were used to characterize the viromes of three principal arboviral vectors, including *Ae. albopictus*, *Cx. tritaeniorhynchus*, and *An. sinensis*, in Jiangxi Province, China’s subtropical monsoon zone (high temperatures and humidity; Table 1) [39]. Research has shown that climatic factors in regions with high temperatures and humidity correlate positively with the density of *Ae. albopictus* populations and increase the risk of spreading diseases such as dengue fever. The natural conditions in Jiangxi Province are conducive to the breeding of mosquitoes such as *Ae. albopictus* and the transmission of vector-borne diseases. Therefore, it is critical to strengthen vector-borne virus surveillance in this region. The aim was to compare the metavirome profiles of these species and to identify potential pathogenic viruses as well as ISVs that may influence vector competence and disease transmission. These findings contribute to understanding the complex interactions between mosquitoes and their viral communities, with implications for arbovirus surveillance and control strategies in Jiangxi Province.

A total of 86 viruses were identified in the nine mosquito pools examined in this study. The majority of these viruses are unclassified (43.02%). The lack of formal taxonomic classification limits our comprehension of the diversity of unclassified viruses circulating in these vector species [40]. The high proportion of unclassified viruses may be related to the inadequacy of the current database. In addition, the lack of reference sequences for several unknown viruses makes annotation difficult, which can result in viruses remaining unannotated and hinder the discovery of other unknown viruses. These viruses can only be accurately named through the continuous refinement of relevant virus databases. Despite having various ecological niches and geographical ranges, the three mosquito species did not exhibit significantly different α-diversity metrics for the 86 viruses (*P* > 0.05). Such a similarity may be related to the environmental factors that mosquitoes were exposed to, host selection, and the adaptability of viruses themselves. Research has shown that the viral composition of mosquitoes, individually and in populations, was closely related to host phylogeny, climate, land use type, and other factors [41]. However, examination of the top 30 viruses from each mosquito pool revealed evident differences in the highly abundant viruses of the three species and formed three distinct genetic branches (Fig. 4B). This tripartite segregation implied the strong host phylosymbiosis of several viruses, where virome configuration mirrored mosquito phylogeny, which indicates codivergence over the past million years [40, 42, 43].

*Solemoviridae*, *Xinmoviridae*, *Phasmaviridae*, *Flaviviridae*, *Rhabdoviridae*, *Peribunyaviridae*, *Orthomyxoviridae*, and unclassified viruses accounted for the highest number of reads in this study. The *Solemoviridae* family is a group of positive-sense single-stranded RNA viruses that infect plants, with genome lengths ranging from four to six kilobases (kb). These viruses are transmitted through mechanical damage, vegetative propagation, insect vectors, or nonbiological pathways such as soil [44].This study identified viruses belonging to the *Solemoviridae* family that were present in all three *Ae. albopictus* pools and in two *Cx. tritaeniorhynchus* pools. The Sichuan mosquito sobemo-like virus and Guangzhou sobemo-like virus were relatively highly abundant in all three *Ae. albopictus* pools. However, none of these viruses were present in the three *Cx. tritaeniorhynchus* pools. Multiple *sobemo-like viruses* were present in *Ae. albopictus*. Several studies, including this one, have detected WSLV4 in this mosquito species [45, 46]. In addition, Guangzhou sobemo-like virus was detected in *Ae. albopictus* from Hainan Island and Cameroon, which further supports its widespread presence in this mosquito species [47, 48]. The other two viruses in the *Solemoviridae* family, *Yongsan sobemo-like virus 1* (YSLV1; JJSD pool, 0.46%) and *Bat sobemovirus* (BSV; NCSD, 13.61%), achieved high read counts in the two *Cx. tritaeniorhynchus* pools. However, they were not detected in any of the three *Ae. albopictus* pools. YSLV1 was shown to be prevalent in *Aedes vexans nipponii* in Korea, likely owing to its high adaptability and capacity for stable replication in mosquito cells [49]. BSV is a virus found in bats, but reports of its presence in mosquitoes are lacking. To date, research primarily focuses on the diversity and distribution of the virus within bat populations [50]. The present study revealed a higher abundance of BSV viruses in the NCSD pool, which deserves further investigation.

The family *Xinmoviridae* comprises negative-sense RNA viruses (9–14 kb genomes) infecting diverse arthropods, though their host range remains poorly defined [51]. Metaviromic profiling revealed a high relative abundance of *Xinmoviridae* in all *An. sinensis* and *Ae. albopictus* pools, with AealbAV dominating *Ae. albopictus* and XCV mainly reported in *An. sinensis*. These two viruses were highly similar and clustered together on a single branch (Fig. 5). Crucially, neither virus was detected in *Cx. tritaeniorhynchus* pools. AealbAV is a mosquito-specific virus widespread in *Ae. albopictus* populations globally. It has been detected in laboratory and field-collected mosquitoes and was distributed across the mosquito life cycle [52]. AealbAV can moderately reduce DENV replication in vitro, and it is a promising tool for future biocontrol measures [53]. Our data confirm AealbAV’s pan-regional dominance in *Ae. albopictus* in Jiangxi. Complete genomes assembled from two pools (GenBank: OR715784 and OR729834) showed minimal divergence (similarity: 99.98%). XCV is a mosquito-specific virus classified within the genus *Anophevirus* and has been first characterized in *An. sinensis* from China [54]. XCV exhibited ubiquitous colonization in *Anopheles* mosquito populations with no documented vertebrate pathogenicity. As a mosquito-specific virus, it may modulate vector competence of those mosquitoes [55, 56]. This study revealed the high abundance of XCV in all three *An. sinensis* pools (GZZH 43.92%, JJZH 1.28%, and NCZH 46.78%), which suggests that XCV may occupy an important ecological niche within these populations.

*Phasmaviridae* comprises enveloped viruses with trisegmented negative-sense RNA genomes (9.7–15.8 kb). These viruses are primarily maintained and transmitted by insects [57]. In this study, *Phasmaviridae* reads dominated all three *An. sinensis* pools (GZZH 55.42%, JJZH 60.25%, and NCZH 50.95%). High read numbers were also recorded in the GZBW (54.28%), GZSD (28.57%), and JJSD (10.64%) sample pools.* Barstukas virus* was the main virus species annotated in the GZBW pool. It has also been detected in *Ae. albopictus* mosquitoes on Hainan Island and is recognized as an environmentally sourced virus [48]. This virus showed a high degree of similarity with other viruses (WMV1 and *Orthophasmavirus aedis*) in the *Phasmaviridae* family (Fig. 5). WMV2 was the predominant virus species identified in *Cx. tritaeniorhynchus* sample pools. WMV1 was the predominant virus species in the three *An. sinensis* pools. Both viruses are ISVs and were first detected in 2015 in *Cx. tritaeniorhynchus* sample pools from Hubei Province, China [54, 58]. WMV2 was also detected in *Cx. tritaeniorhynchus* pools from Yunnan and Shandong Provinces in China and from Japan [59–61]. In addition, WMV2 is widely found in other mosquitoes, such as *Armigeres subalbatus*, *An. sinensis*, *Cx. quinquefasciatus*, and *Ae. aegypti* [59, 62]. WMV1 is widely found in *Cx. tritaeniorhynchus* and *Anopheles* mosquito populations but has not been reported in other mosquito species [19, 55]. This research supports this assertion.

This study identified six distinct viruses from the *Flaviviridae* family in the nine mosquito pools [*Aedes* flavivirus (AEFV), *dengue virus 3* (DENV-3), GUAPV, QBV, *Culex tritaeniorhynchus* *flavi-like virus*, and *Kamiti River virus* (KRV)]. In total, five viruses were annotated to the NCBW pool (except for *Culex tritaeniorhynchus* *flavi-like virus*), but the number of reads for the other four viruses was low, except for AEFV (4.88%). AEFV is an ISVs first isolated from* Ae. albopictus* and *Ae. flavopictus* in Japan [63]. In several cases, these ISVs can interfere with pathogenic flavivirus infections, which suggests potential applications in disease control [63]. DENV-3 is one of the four DENV serotypes [64] that triggered several large-scale epidemics worldwide [65–67]. This work revealed that the DENV-3 was present in the NCBW and GZBW pools, which suggests that the virus is being transmitted by *Ae. albopictus* in Jiangxi Province and thus needs further research. QBV is an ISV that was first identified in *Culex* mosquitoes collected in Quang Binh, Vietnam, in 2002 [68]. A high rate of QBV was detected in *Cx. tritaeniorhynchus* populations, whereas a relatively low rate was observed in *An. sinensis* [69–72]. It was annotated to all three *Cx. tritaeniorhynchus* pools and the NCZH pool in this study. QBV isolated from Guizhou Province formed a unique branch with the Vietnamese strain but was closely related to QBV isolates from other regions of China. This genetic diversity may be related to the virus’s adaptability and transmissibility [73]. Other viruses, such as GUAPV, *Culex tritaeniorhynchus* *flavi-like virus*, and KRV, have not been fully clarified in terms of their transmission mechanisms in mosquitoes. However, these viruses should be further studied to assess their pathogenic potential and their potential public health risks is also worth exploring further [71, 74, 75].

*Rhabdoviridae* comprises enveloped viruses with negative-sense single-stranded (ss)RNA genomes (10–16 kb), and it demonstrates exceptional host breadth [76]. The *Rhabdoviridae* virus annotated to two *Ae. albopictus* pools (JJBW and NCBW) was Piry virus (PIRYV). This virus belongs to the *Vesiculovirus* genus, which is described as a potential human pathogen; it was first isolated from *Philander opossum* captured in the state of Pará in northern Brazil [77]. This virus has been detected in cell cultures of *Ae. aegypti* and *Ae. albopictus*, which further confirms its ability to grow within these mosquitoes [78]. Although PIRYV has not shown direct pathogenicity to humans, its continued transmission in mosquitoes and potential infectivity in mammals remain a cause for concern. The virus identified in the *Cx. tritaeniorhynchus* (NCSD and GZSD) and *An. sinensis* (NCZH) pools was the CTRV, which was also isolated from *Cx. tritaeniorhynchus* subspecies [79]. This virus is widespread in the natural populations and cell lines of *Cx. tritaeniorhynchus*. It is primarily transmitted by mosquitoes and does not cause diseases in vertebrates [80, 81]. The detection of CTRV in this study further indicates its presence in the natural populations of *Cx. tritaeniorhynchus*. CTRV infection may alter the physiological state of mosquitoes, which affects the transmission efficiency of JEV [80]. Furthermore, the presence of CTRV in a sample pool of *An. sinensis* in Nanchang implies its potential to adapt to various environments and hosts.

*Peribunyaviridae* encompasses several genera of viruses distributed worldwide [82]. In this study, *Culex pseudovishnui* *bunya-like virus* (CPBV) was annotated in high abundance in a *Cx. tritaeniorhynchus* pool (JJSD 17.77%), and WVD virus was annotated to the *An. sinensis* pool (JJZH 0.53%). CPBV is an ISV that was first identified in *Cx. pseudovishnui* [61]; its distribution, biology, and potential public health significance in mosquito species are unknown. WVD, a pathogenic virus that was first isolated in humans, is highly homologous to the Wyeomyia mosquito virus [38, 83]. In this study, a relatively high abundance of WVD virus was annotated to the JJZH pool, which provides a new opportunity for an in-depth investigation of the ecological properties of this virus in mosquitoes and its transmission cycle and potential public health significance.

*Orthomyxoviridae* viruses are a group of viruses that have important implications for medicine and public health. For example, influenza viruses (A and B) from this family are the most likely to cause seasonal epidemics and occasional pandemics in humans [84]. This study annotated the AOMV-2 and *Wuhan mosquito virus* *5* (WMV5) from *Orthomyxoviridae* family to the *Ae. albopictus* (JJBW 61.41%) and *An. sinensis* (JJZH 3.50%) pools, respectively. AOMV-2 was detected with high prevalence in laboratory and field strains of *Ae. albopictus* populations [85–87], and a strong association with host cell nuclei was found [88]. In this study, AOMV-2 was also detected in high abundance in the *Ae. albopictus* population in Jiujiang, which warranted further investigation to explore the intrinsic and extrinsic factors contributing to its high relative abundance. WMV5 was first detected in *Cx. tritaeniorhynchus* in Hubei Province, China, in 2015 [54]. However, it is not an ISV and was found to be distinct from WMV1 and WMV2. Further research is needed to investigate the transmission patterns of WMV5 in *An. sinensis* populations in natural environments.

Unclassified viruses constituted 43.02% (37/86) of the total virome, and they exhibited a relatively high abundance in four mosquito pools (Fig. 3). High Island virus and Usinis virus were the most frequently detected viruses in *Ae. albopictus*; *Hubei mosquito virus 2* was the most frequently detected virus in *Cx. tritaeniorhynchus*; and *Hubei reo-like virus 12* was the most frequently detected virus in *An. sinensis*. These viruses were also previously documented in diverse mosquito vectors globally [23, 81, 88, 89]. The abundance and diversity of unclassified viruses represent the bulk of the planet’s undescribed genetic diversity and are key to understanding viral evolution, host ranges, ecological distributions, and origins [58, 90]. Their discovery has provided a new perspective on the origin of viruses and their role in the evolutionary history of hosts. Further research into the biological characteristics, pathogenicity, and effects on vector competence of these unclassified viruses would be worthwhile.

## Conclusions

This study used NGS technology to perform metavirome sequencing on *Ae. albopictus*, *Cx. tritaeniorhynchus*, and *An. sinensis*, which were collected in Jiangxi Province, China. A total of 86 viruses were annotated, and they included 49 known viruses belonging to 19 viral families and 37 unclassified viruses. The latter accounted for the highest proportion. No significant difference was observed in the diversity of the 86 viruses among the three mosquito species. However, the most frequently detected viruses of the three species showed distinct differences. The most frequently detected viruses of the *Ae. albopictus* population include the Sichuan mosquito sobemo-like virus and Guangzhou sobemo-like virus (*Solemoviridae*), AealbAV (*Xinmoviridae*), *Barstukas virus* (*Phasmaviridae*), AEFV (*Flaviviridae*), PIRYV (*Rhabdoviridae*), and the most abundant High Island virus and Usinis virus. The most frequently detected viruses of the *Cx. tritaeniorhynchus* population include the YSLV1 and BSV (*Solemoviridae*), WMV2 (*Phasmaviridae*), QBV (*Flaviviridae*), CTRV (*Rhabdoviridae*), CPBV, and *Hubei mosquito virus 2* (*Peribunyaviridae*). The most frequently detected viruses of the *An. sinensis* population comprise the XCV (*Xinmoviridae*), WMV1 (*Phasmaviridae*), WMV5 (*Orthomyxoviridae*), and the *Hubei reo-like virus 12*. This study utilized bioinformatics analysis to further assemble and analyze several of these viruses. It explored their potential biological significance and evolutionary relationships through a combination of de novo genome assembly and phylogenetic reconstruction. This provides foundational data for further research into the interactions between these viruses and mosquitoes. Although this study allowed for the extensive screening of viral diversity within mosquito sample pools, notable limitations that require refinement were still noted. First, short-read sequencing technology struggles to accurately assemble complex viral genomes, which has a particular effect on variant analysis. Second, the lack of standardization in bioinformatics analysis workflows and incomplete databases restricted the annotation and functional prediction of highly variable or novel viruses. Furthermore, this method only detects the presence of viruses, rather than proves mosquito vector capacity, vector competence, or viral biological properties directly. Subsequent traditional virus isolation and experimental validation are required to clarify the ecological and epidemiological significance of the findings.

## Supplementary Information

Supplementary material 1. Additional file 1: Table S1. Data output summary of *Ae. albopictus, Cx. tritaeniorhynchus* and *An. sinensis* pools. Additional file 2: Table S2. Virus composition and read numbers in *Ae. albopictus, Cx. tritaeniorhynchus *and *An. sinensis* pools

## Data Availability

The raw sequencing data were deposited in the National Library of Medicine (NIH)-JJBW: PRJNA1162562, SAMN43816587, SRR30716719; NCBW: PRJNA1162665, SAMN43818466, SRR30718056; GZBW: PRJNA1162937, SAMN43832959, SRR30730294; JJSD: PRJNA1162936, SAMN43832956, SRR30730374; NCSD: PRJNA1162936, SAMN43832957, SRR30730373; GZSD: PRJNA1162936, SAMN43832958, SRR30730372; JJZH: PRJNA1163432, SAMN4385066, SRR30754617; NCZH: PRJNA1163432, SAMN43850667, SRR30754616; and GZZH: PRJNA1163432, SAMN43850668, SRR30754615.

## Abbreviations

AealbAV: Aedes albopictus anphevirus
AEFV: Aedes flavivirus
AOMV-2: Aedes orthomyxo-like virus 2
BSV: Bat sobemovirus
CHIKV: Chikungunya virus
CPBV: Culex pseudovishnui bunya-like virus
CTRV: Culex tritaeniorhynchus rhabdovirus
DENV: Dengue virus
DNBs: DNA nanoballs
EILV: Eilat virus
GUAPV: Guapiacu virus
ISVs: Insect-specific viruses
JEV: Japanese encephalitis virus
KRV: Kamiti river virus
NHUV: Nhumirim virus
NGS: Next-generation sequencing
PIRYV: Piry virus
QBV: Quang Binh virus
RdRp: RNA-dependent RNA polymerase
SHTV: Shinobi tetravirus
WMV1: Wuhan mosquito virus 1
WMV2: Wuhan mosquito virus 2
WMV5: Wuhan mosquito virus 5
WNV: West Nile virus
WSLV4: Wenzhou sobemo-like virus 4
WVD: Wyeomyia virus strain Darien
XCV: Xincheng mosquito virus
YFV: Yellow fever virus
YSLV1: Yongsan sobemo-like virus 1
ZIKV: Zika virus
