# Supplementary material for: Differences in metavirome among Aedes albopictus, Culex tritaeniorhynchus, and Anopheles sinensis in Jiangxi Province, China
Source: Parasit Vectors. 2026 Jan 5;19:14. doi: 10.1186/s13071-025-07195-y (PMC12781663; doi:10.1186/s13071-025-07195-y)
Supplement: Supplementary file 1 — Supplementary material 1. Additional file 1: Table S1. Data output summary of Ae. albopictus, Cx. tritaeniorhynchus and An. sinensis pools. Additional file 2: Table S2. Virus composition and read numbers in Ae. albopictus, Cx. tritaeniorhynchus and An. sinensis pools [file 13071_2025_7195_MOESM1_ESM.doc]

Table S1 Data output summary of *Ae. albopictus*, *Cx. tritaeniorhynchus* and *An. sinensis* pools

|  | RawReads  (M) | RawBases  (G) | RawData_Q30  (%) | RawData_GC  (%) | CleanReads  (M) | CleanBases  (G) | CleanData_Q30  (%) | CleanData_GC  (%) |
| --- | --- | --- | --- | --- | --- | --- | --- | --- |
| NCBW | 179.96 | 26.99 | 97.95 | 52.00 | 179.86 | 22.31 | 97.98 | 52.23 |
| NCSD | 204.51 | 30.68 | 98.72 | 51.06 | 204.41 | 23.42 | 98.75 | 50.97 |
| NCZH | 163.87 | 24.58 | 97.75 | 51.83 | 163.81 | 19.80 | 97.94 | 51.63 |
| JJBW | 222.01 | 33.30 | 98.03 | 53.39 | 221.94 | 26.60 | 98.15 | 53.65 |
| JJSD | 172.86 | 25.93 | 98.63 | 52.49 | 172.80 | 19.28 | 98.69 | 52.47 |
| JJZH | 180.22 | 27.03 | 98.19 | 51.71 | 180.17 | 20.86 | 98.31 | 51.64 |
| GZBW | 152.75 | 22.91 | 97.63 | 52.61 | 152.69 | 19.08 | 97.71 | 52.68 |
| GZSD | 187.42 | 28.11 | 98.31 | 49.01 | 187.32 | 23.20 | 98.33 | 48.35 |
| GZZH | 198.26 | 29.74 | 98.27 | 51.87 | 198.16 | 23.73 | 98.41 | 51.62 |

| Table S2 Virus composition and read numbers in Ae. albopictus, Cx. tritaeniorhynchus and An. sinensis pools | | | | | | | | | | |
| --- | --- | --- | --- | --- | --- | --- | --- | --- | --- | --- |
| Family | Species | GZBW | GZSD | GZZH | JJBW | JJSD | JJZH | NCBW | NCSD | NCZH |
| *Bromoviridae* | *Citrus leaf rugose virus* | 0 | 0 | 0 | 39 | 0 | 0 | 0 | 0 | 0 |
| *Bromoviridae* | *Citrus variegation virus* | 0 | 0 | 0 | 16 | 0 | 0 | 0 | 0 | 0 |
| *Chuviridae* | *Culex mosquito virus 4* | 0 | 0 | 0 | 0 | 137412 | 0 | 0 | 0 | 0 |
| *Chuviridae* | *Gurupi chuvirus-like 2* | 0 | 0 | 0 | 0 | 0 | 0 | 283 | 0 | 0 |
| *Chuviridae* | *Gurupi chuvirus-like 1* | 0 | 0 | 0 | 0 | 0 | 0 | 14 | 0 | 0 |
| *Flaviviridae* | *dengue virus type 3* | 1765 | 0 | 0 | 0 | 0 | 0 | 17591 | 0 | 0 |
| *Flaviviridae* | *Guapiacu virus* | 706 | 0 | 0 | 0 | 0 | 0 | 1136 | 0 | 0 |
| *Flaviviridae* | *Mosquito flavivirus* | 0 | 7916 | 0 | 0 | 131 | 0 | 18 | 0 | 0 |
| *Flaviviridae* | *Quang Binh virus* | 0 | 21352 | 0 | 0 | 3706 | 0 | 16 | 17231 | 416095 |
| *Flaviviridae* | *Kamiti River virus* | 0 | 0 | 0 | 0 | 0 | 0 | 52 | 0 | 0 |
| *Flaviviridae* | *Aedes flavivirus* | 0 | 0 | 0 | 0 | 0 | 0 | 221179 | 0 | 0 |
| *Flaviviridae* | *Culex tritaeniorhynchus flavi-like virus* | 0 | 0 | 0 | 0 | 0 | 0 | 0 | 2893 | 0 |
| *Iflaviridae* | *Isahaya Culex iflavirus* | 0 | 0 | 0 | 0 | 9393 | 0 | 0 | 0 | 0 |
| *Luteoviridae* | *Culex associated luteo like virus* | 0 | 0 | 0 | 0 | 0 | 0 | 0 | 0 | 13 |
| *Narnaviridae* | *Narnaviridae* sp*.* | 0 | 6590 | 0 | 0 | 0 | 0 | 0 | 0 | 0 |
| *Orthomyxoviridae* | *Wuhan Mosquito Virus 6* | 0 | 4497 | 0 | 0 | 13 | 0 | 0 | 0 | 0 |
| *Orthomyxoviridae* | *Aedes orthomyxo-like virus 2* | 0 | 0 | 0 | 23090364 | 0 | 0 | 0 | 0 | 0 |
| *Orthomyxoviridae* | *Orthomyxoviridae* sp*.* | 0 | 0 | 0 | 0 | 3116 | 0 | 0 | 0 | 0 |
| *Orthomyxoviridae* | *Pyongtaek Culex Orthomyxovirus* | 0 | 0 | 0 | 0 | 3707 | 0 | 0 | 0 | 0 |
| *Orthomyxoviridae* | *Wuhan Mosquito Virus 4* | 0 | 0 | 0 | 0 | 8103 | 0 | 0 | 0 | 0 |
| *Orthomyxoviridae* | *Wuhan Mosquito Virus 5* | 0 | 0 | 0 | 0 | 0 | 2479778 | 0 | 0 | 6678 |
| *Partitiviridae* | *Culex pseudovishnui partitivirus* | 0 | 85 | 0 | 0 | 0 | 0 | 0 | 0 | 0 |
| *Peribunyaviridae* | *Culex pseudovishnui bunya-like virus* | 0 | 0 | 0 | 0 | 1259504 | 0 | 0 | 0 | 0 |
| *Peribunyaviridae* | *Xinzhou Mosquito Virus* | 0 | 0 | 0 | 0 | 0 | 62043 | 0 | 0 | 0 |
| *Peribunyaviridae* | *Wyeomyia virus strain Darien* | 0 | 0 | 0 | 0 | 0 | 372706 | 0 | 0 | 0 |
| *Peribunyaviridae* | *Qingnian Mosquito Virus* | 0 | 0 | 0 | 0 | 0 | 0 | 0 | 235 | 0 |
| *Phasmaviridae* | *Barstukas virus* | 2298601 | 0 | 0 | 194054 | 0 | 0 | 0 | 0 | 0 |
| *Phasmaviridae* | *Wuhan Mosquito Virus 2* | 0 | 107989 | 0 | 0 | 714023 | 0 | 0 | 14 | 0 |
| *Phasmaviridae* | *Culex phasma-like virus* | 0 | 24 | 0 | 0 | 0 | 0 | 0 | 0 | 0 |
| *Phasmaviridae* | *Orthophasmavirus aedis* | 0 | 1955 | 0 | 0 | 40059 | 0 | 0 | 0 | 0 |
| *Phasmaviridae* | *Wuhan mosquito virus 1* | 0 | 0 | 4372765 | 0 | 0 | 33029326 | 0 | 0 | 24564077 |
| *Phasmaviridae* | *Orthophasmavirus wuhanense* | 0 | 0 | 0 | 0 | 0 | 9652051 | 0 | 0 | 420440 |
| *Phenuiviridae* | *Okutama tick virus* | 0 | 10594 | 0 | 0 | 0 | 0 | 0 | 0 | 0 |
| *Qinviridae* | *Fitzroy Crossing qinvirus 1* | 0 | 0 | 0 | 0 | 8 | 0 | 0 | 10 | 0 |
| *Rhabdoviridae* | *Culex tritaeniorhynchus rhabdovirus* | 0 | 68462 | 0 | 0 | 0 | 0 | 0 | 278374 | 662981 |
| *Rhabdoviridae* | Piry virus | 0 | 0 | 0 | 17401 | 0 | 0 | 5251 | 0 | 0 |
| *Secoviridae* | *Broad bean wilt virus 2* | 0 | 0 | 0 | 0 | 0 | 0 | 419 | 0 | 0 |
| *Solemoviridae* | Sichuan mosquito sobemo-like virus | 20742 | 0 | 0 | 54734 | 0 | 0 | 566577 | 0 | 0 |
| *Solemoviridae* | Guangzhou sobemo-like virus | 77635 | 0 | 0 | 41889 | 0 | 0 | 431963 | 0 | 0 |
| *Solemoviridae* | *Yongsan sobemo-like virus 1* | 0 | 0 | 0 | 0 | 32704 | 0 | 0 | 0 | 0 |
| *Solemoviridae* | *Bat sobemovirus* | 0 | 0 | 0 | 0 | 0 | 0 | 0 | 1827313 | 119 |
| *Tombusviridae* | *Yongsan tombus-like virus 1* | 0 | 0 | 0 | 0 | 0 | 0 | 133624 | 0 | 0 |
| *Totiviridae* | *Pisingos virus* | 46 | 0 | 0 | 0 | 0 | 0 | 78 | 0 | 0 |
| *Totiviridae* | *Culex vishnui subgroup totivirus* | 0 | 0 | 0 | 0 | 48 | 0 | 0 | 0 | 0 |
| *Totiviridae* | *XiangYun toti-like virus 5* | 0 | 0 | 0 | 0 | 837 | 0 | 0 | 0 | 0 |
| *Tymoviridae* | *Culex pseudovishnui tymo-like virus* | 0 | 0 | 0 | 0 | 192 | 0 | 0 | 0 | 0 |
| *Xinmoviridae* | *Aedes albopictus anphevirus* | 25036 | 0 | 0 | 22899 | 0 | 0 | 55424 | 0 | 0 |
| *Xinmoviridae* | *Xincheng Mosquito Virus* | 0 | 0 | 3465592 | 0 | 0 | 905656 | 0 | 0 | 22943093 |
| *Xinmoviridae* | *Anphevirus* sp*.* | 0 | 0 | 0 | 0 | 0 | 9294062 | 0 | 0 | 0 |
| Unclassified | Usinis virus | 1609679 | 0 | 0 | 2493602 | 0 | 0 | 1581222 | 0 | 0 |
| Unclassified | *Longgang virus* | 135 | 0 | 0 | 9988 | 0 | 0 | 8133 | 0 | 0 |
| Unclassified | *Wenzhou sobemo-like virus 4* | 16790 | 0 | 0 | 210388 | 0 | 0 | 42495 | 0 | 0 |
| Unclassified | High Island virus | 141828 | 0 | 0 | 11407601 | 0 | 0 | 949158 | 0 | 0 |
| Unclassified | *Guato virus* | 251 | 0 | 0 | 1871 | 0 | 0 | 2716 | 0 | 0 |
| Unclassified | *Kaiowa virus* | 94 | 0 | 0 | 0 | 0 | 0 | 151 | 0 | 0 |
| Unclassified | *Aedes binegev-like virus 1* | 14 | 0 | 0 | 0 | 0 | 0 | 109 | 0 | 0 |
| Unclassified | *Bacteriophage* sp*.* | 0 | 5 | 0 | 0 | 0 | 0 | 0 | 0 | 0 |
| Unclassified | *Broome luteo-like virus 1* | 0 | 118007 | 0 | 0 | 0 | 0 | 0 | 0 | 0 |
| Unclassified | *Pine Lake virus* | 0 | 985 | 0 | 0 | 0 | 0 | 0 | 0 | 0 |
| Unclassified | *XiangYun mono-chu-like virus 5* | 0 | 40 | 0 | 0 | 0 | 0 | 0 | 0 | 0 |
| Unclassified | *Yongsan picorna-like virus 2* | 0 | 13905 | 0 | 0 | 0 | 0 | 0 | 0 | 0 |
| Unclassified | *Mononegavirales* sp*.* | 0 | 19815 | 0 | 0 | 0 | 0 | 0 | 2616 | 0 |
| Unclassified | *XiangYun narna-levi-like virus 13* | 0 | 28 | 0 | 0 | 0 | 0 | 0 | 0 | 0 |
| Unclassified | *Riboviria* sp*.* | 0 | 2612 | 11025 | 0 | 0 | 12282 | 0 | 0 | 2233 |
| Unclassified | *XiangYun bunya-arena-like_virus 14* | 0 | 0 | 14320 | 0 | 0 | 0 | 0 | 0 | 0 |
| Unclassified | *Hubei reo-like virus 12* | 0 | 0 | 9710 | 0 | 0 | 15027618 | 0 | 0 | 23358 |
| Unclassified | *Hubei virga-like virus 23* | 0 | 0 | 17127 | 0 | 0 | 0 | 0 | 0 | 0 |
| Unclassified | *Gentian Kobu-sho-associated virus* | 0 | 0 | 399 | 0 | 0 | 0 | 0 | 0 | 0 |
| Unclassified | *Shinobi tetravirus* | 0 | 0 | 0 | 41018 | 0 | 0 | 511430 | 0 | 0 |
| Unclassified | *Aedes binegev-like virus 2* | 0 | 0 | 0 | 12737 | 0 | 0 | 0 | 0 | 0 |
| Unclassified | *San Gabriel mononegavirus* | 0 | 0 | 0 | 3217 | 0 | 0 | 1519 | 0 | 0 |
| Unclassified | *Netjeret virus* | 0 | 0 | 0 | 594 | 0 | 0 | 0 | 0 | 0 |
| Unclassified | *Wolbachia phage WO* | 0 | 0 | 0 | 8 | 0 | 0 | 0 | 0 | 0 |
| Unclassified | *Hubei mosquito virus 2* | 0 | 0 | 0 | 0 | 4980387 | 0 | 0 | 11292909 | 1601 |
| Unclassified | *Zhejiang mosquito virus 3* | 0 | 0 | 0 | 0 | 226 | 0 | 0 | 0 | 0 |
| Unclassified | *XiangYun bunya-arena-like virus 4* | 0 | 0 | 0 | 0 | 27 | 0 | 0 | 0 | 0 |
| *Unclassified* | *Culex pseudovishnui negev-like virus* | 0 | 0 | 0 | 0 | 33039 | 0 | 0 | 0 | 0 |
| *Unclassified* | *XiangYun partiti-picobirna-like virus 7* | 0 | 0 | 0 | 0 | 0 | 626 | 0 | 0 | 0 |
| *Unclassified* | *Caudoviricetes* sp*.* | 0 | 0 | 0 | 0 | 0 | 0 | 514 | 0 | 0 |
| *Unclassified* | *Ormpussen virus* | 0 | 0 | 0 | 0 | 0 | 0 | 34 | 0 | 0 |
| *Unclassified* | *Siphoviridae sp. ctWWc42* | 0 | 0 | 0 | 0 | 0 | 0 | 848 | 0 | 0 |
| *Unclassified* | *Aedes aegypti To virus 2* | 0 | 0 | 0 | 0 | 0 | 0 | 41 | 0 | 0 |
| *Unclassified* | *Aedes aegypti virga-like virus* | 0 | 0 | 0 | 0 | 0 | 0 | 193 | 0 | 0 |
| *Unclassified* | *Arceuthobium sichuanense virus 3* | 0 | 0 | 0 | 0 | 0 | 0 | 400 | 0 | 0 |
| *Unclassified* | *Wenzhou sobemo-like virus 3* | 0 | 0 | 0 | 0 | 0 | 0 | 0 | 6238 | 121 |
| *Unclassified* | *Pyongtaek Culex sobemo-like virus* | 0 | 0 | 0 | 0 | 0 | 0 | 0 | 473 | 64 |
